# A Case Report Demonstrating Preservation of Vestibular Receptor Function after Transcochlear Removal of an Intracochlear Schwannoma with Extension to the Fundus of the Internal Auditory Canal

**DOI:** 10.3390/jcm13123373

**Published:** 2024-06-07

**Authors:** Stefan K. Plontke, Francesco P. Iannacone, Udo Siebolts, Beatrice Ludwig-Kraus, Sabrina Kösling, Luise Wagner

**Affiliations:** 1 Department of Otorhinolaryngology, Head & Neck Surgery, Martin Luther University Halle-Wittenberg, 06120 Halle (Saale), Germany; luise.wagner@uk-halle.de; 2 Department of Neuroscience DNS, Otolaryngology Section, University of Padova, 35122 Padova, Italy; 3 Institute of Pathology, Martin Luther University Halle-Wittenberg, 06112 Halle (Saale), Germany; 4 Institute of General Pathology and Pathological Anatomy, Molecular Pathology Diagnostics, University Hospital, 50937 Cologne, Germany; 5 Department of Laboratory Medicine, Central Laboratory, University Hospital Halle, 06120 Halle (Saale), Germany; 6 Department of Radiology, Martin Luther University Halle-Wittenberg, 06120 Halle (Saale), Germany

**Keywords:** intracochlear schwannoma, intralabyrinthine schwannoma, vestibular schwannoma, vertigo

## Abstract

Preservation of function is an important goal during surgical management of cochleovestibular schwannomas. We here demonstrate the relief of vertigo and the preservation of function of all five vestibular receptors after removal of an intracochlear schwannoma with extension to the fundus of the internal auditory canal. A 61-year-old male with a five-year history of left-sided deafness, tinnitus, vertigo attacks, and an MRI consistent with an intracochlear schwannoma with limited extension through the modiolus to the fundus of the internal auditory canal (IAC) underwent transcanal, transcochlear total tumor removal and—due to a cerebrospinal fluid leak from the fundus of the IAC—revision surgery with lateral petrosectomy and blind sac closure of the external auditory canal. Despite complete removal of the cochlear partition of the inner ear (total cochlectomy), the patient’s vestibular receptors remained functional, and the vertigo symptoms disappeared. These results show that vestibular labyrinthine function may not only be preserved after partial or subtotal cochlectomy but also after complete cochlear removal. This further confirms the vestibular labyrinth’s robustness and encourages surgical management of transmodiolar schwannomas with limited extension to the fundus of the IAC.

## 1. Introduction

The management strategies of cochleovestibular schwannomas involve observation, radiotherapy, and microsurgery. Preservation of neurological function is an important goal during surgical management. The three most common microsurgical approaches used to remove vestibular schwannomas of the internal auditory canal (IAC) and/or the cerebello-pontine angle (CPA) are the translabyrinthine and the retrosigmoid approaches, that can be used even for the resection of large tumors, and the middle fossa approach for smaller tumors in the IAC and only limited extension to the CPA [1]. Further approaches are the retrolabyrinthine approach, which is especially suited for medially located tumors [2], and the more recently suggested exclusively endoscopic and expanded transcanal transpromontorial or transcanal infrapromontorial approaches for vestibular schwannoma [3,4,5]. The retrosigmoid, the middle fossa, and the retrolabyrinthine approaches offer the possibility of hearing preservation, while destruction of the inner ear structures in the other approaches inherently sacrifices hearing function with anecdotal exceptions [6,7]. If the cochlea and the cochlear nerve can be preserved, hearing can be rehabilitated with cochlear implants (CIs) [8].

For inner ear schwannomas (IES), which are rare, benign tumors located in the periphery of the eighth cranial nerve, treatment depends on tumor characteristics such as size, location, and growth behavior, on clinical symptoms, and on the desired outcome, e.g., in terms of tumor control, vestibular symptoms, and hearing rehabilitation with CIs [9]. Surgical resection is preferred to observation for patients with vestibular symptoms such as dizziness and vertigo [10] and for patients with intended hearing rehabilitation with cochlear implants [11,12]. In cases of a solely intracochlear location of the inner ear schwannoma, observation likely complicates management in the future due to possible growth into the vestibular labyrinth and/or through the modiolus into the internal auditory canal (IAC). Inner ear schwannomas that extend to the IAC at the time of diagnosis represent a greater challenge for treatment than tumors that are confined to the inner ear [9,13,14]. Although resection of an intracochlear tumor bears the risk of postoperative vestibular receptor dysfunction with dizziness and vertigo, it has been shown that vestibular receptor function can be preserved in most cases after partial or subtotal cochlectomy for removal of intracochlear schwannomas [15]. Complete surgical resection of intracochlear tumors extending through the modiolus into the IAC, through a transcochlear approach or complete cochlectomy, represents an even more extensive trauma to the inner ear, with an assumed greater risk of damage to the vestibular labyrinth, although no data are available to date.

We here demonstrate the relief of vertigo attacks and the preservation of function of all five vestibular receptors even after transcochlear tumor removal of an intracochlear schwannoma with extension to the fundus of the internal auditory canal, i.e., after complete cochlear resection.

## 2. Patient Information and Clinical Findings

A 61-year-old European male was referred to our university center with a five-year history of left-sided deafness and tinnitus and attacks of spinning vertigo accompanied by nausea and vomiting. The vertigo attacks had led to several hospital admissions. The patient was otherwise healthy. The clinical otorhinolaryngologic examination including ear microscopy was normal.

## 3. Diagnostic Assessment and Interpretation

Pure tone audiometry showed no measurable threshold on the left and mild sensorineural hearing loss (PTA average of 500, 1000, 2000, and 4000 Hz: 16.25 dB HL) on the right side. Speech intelligibility could not be tested due to a language barrier. Auditory steady state responses (ASSRs) showed no potentials on the left side. The promontory test led to audible perception for 50 Hz, 100 Hz, and 200 Hz. No perception was evoked for 400 Hz.

**Figure 1:**
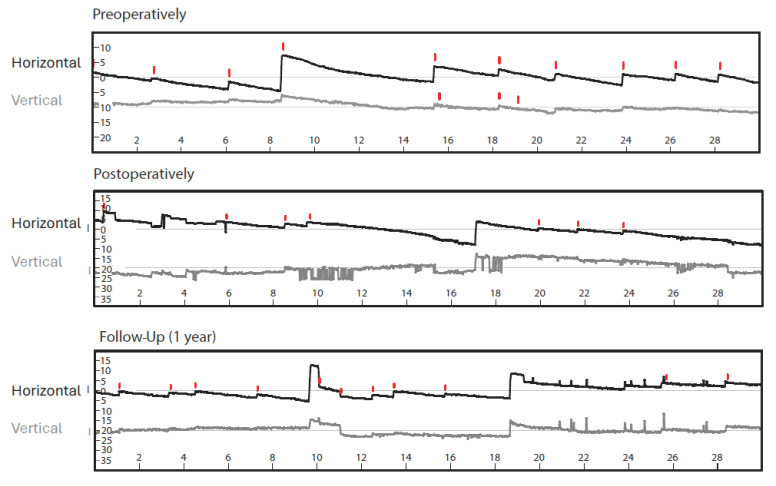
Preoperative videonystagmography showed isolated spontaneous nystagmus to the right, which showed no relevant change postoperatively and after 1 year follow-up. The red lines in horizontal plane mark nystagmus to the right, in vertical plane they symbolize upward movement. The *x*-axis shows time in seconds.

**Figure 2:**
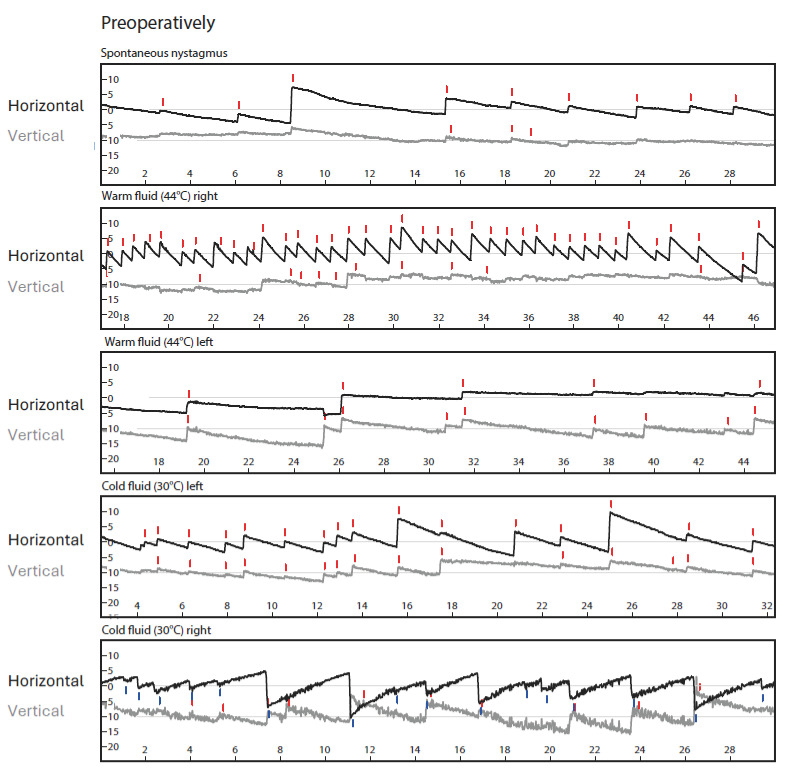
Preoperative videonystagmography showed asymmetry in the caloric excitability favoring the right side. Postoperative caloric testing could not be performed due to blind sac closure of the ear canal. In horizontal plane the red lines mark nystagmus to the right, blue lines to the left. In vertical plane the red lines symbolize upward movement. The *x*-axis shows time in seconds.

Videonystagmography (VNG) showed isolated spontaneous nystagmus to the right (Figure 1) and asymmetry in the caloric excitability favoring the right side (Figure 2).

**Figure 3:**
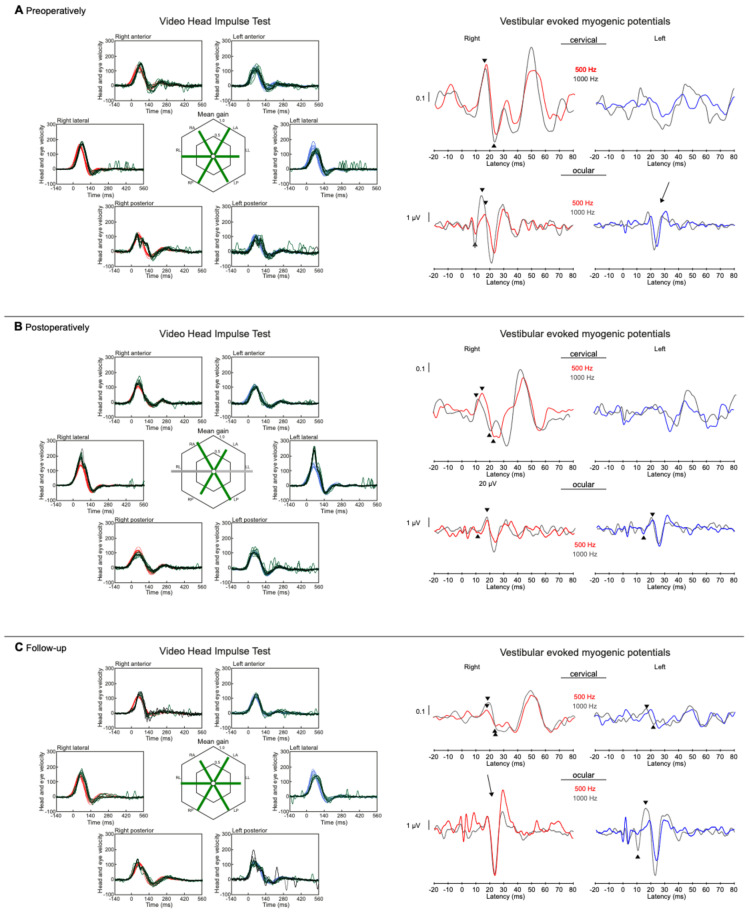
Results of vHIT and bone conducted VEMPs. On the left, the vHIT results with eye velocity in black and head velocity in blue and red are shown. The preoperative (**A**) and 1-year follow-up measurements (**C**) demonstrate physiological responses. The higher lateral gains at (**B**) one week after surgical tumor removal can be interpreted as artifacts due to movements (slipping) of the vHIT glasses. On the right, the VEMPs are plotted with positive values upwards. The 1000 Hz response is plotted in grey, 500 Hz in red or blue corresponding to the side. Cervical VEMPs are plotted in relation to muscular tension. A recovery of ocular VEMPs can be seen at the 1-year follow-up (**C**). Triangles mark minimum and maximum of vestibular evoked potentials. Some non-vestibular evoked potentials (left (**A**) and right (**C**)) can be found (marked with arrows).

The Video Head Impulse Test (vHIT, GN Otometrics, Taastrup, Denmark) showed regular vestibule ocular reflexes in all planes (gains: lateral right: 1.07, left: 0.85; anterior right: 0.96, left: 1.09; and posterior right: 1.01 and left: 0.96) (Figure 3A, left).

Bone-conducted ocular and cervical evoked myogenic potentials (oVEMPs, cVEMPs, Eclipse recording system, Interacoustics A/S, Middlefart, Denmark; B81 transducer) at 500 Hz and 1000 Hz showed clear potentials for the right side and no potentials with vestibular origin on the left side (Figure 3A, right).

**Figure 4:**
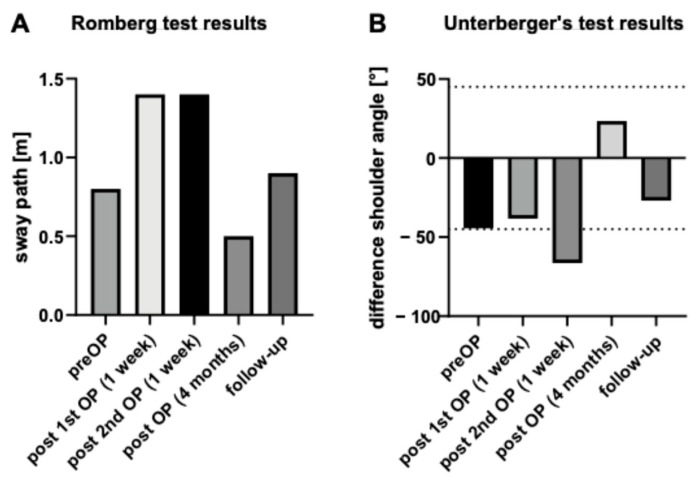
Romberg test results (**A**) showing a slight decrease in stability immediately after surgery but fast recovery to preoperative results or even better. The Unterberger’s test (**B**) was within the physiological limit of 50° during all measurements apart from one week after second surgery.

Romberg’s test was negative (normal) and the Unterberger’s stepping test showed a slight deviation to the left (Figure 4).

**Figure 5:**
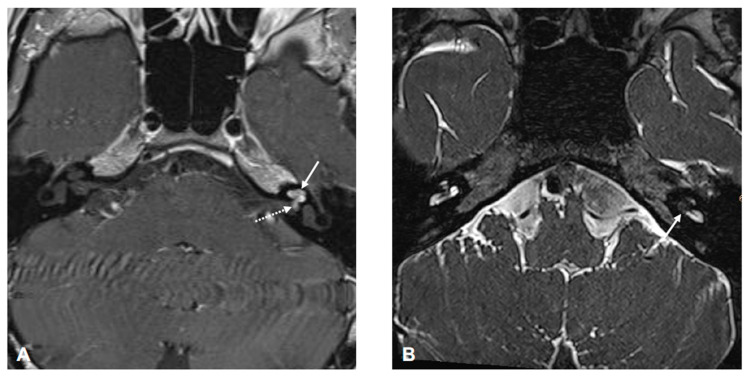
MRI (axial images: (**A**) T1-weighted with contrast medium, (**B**) T2-weighted) showing a left-sided tumor in the apical, middle (arrow in (**A**)), and approximately half of the basal (arrow in **B**) cochlear turn with circumscribed spread via the modiolus and cochlear aperture into fundus of the internal auditory canal (dotted arrow in (**A**)).

Magnetic resonance imaging (MRI) five years after initial symptoms showed a contrast-enhancing lesion in the apical, middle, and partially in the basal cochlear turn with circumscribed spread via the modiolus and cochlear aperture into the fundus of the internal auditory canal consistent with an inner ear schwannoma with limited transmodiolar extension into the fundus of the IAC (Figure 5).

## 4. Intervention

**Figure 6:**
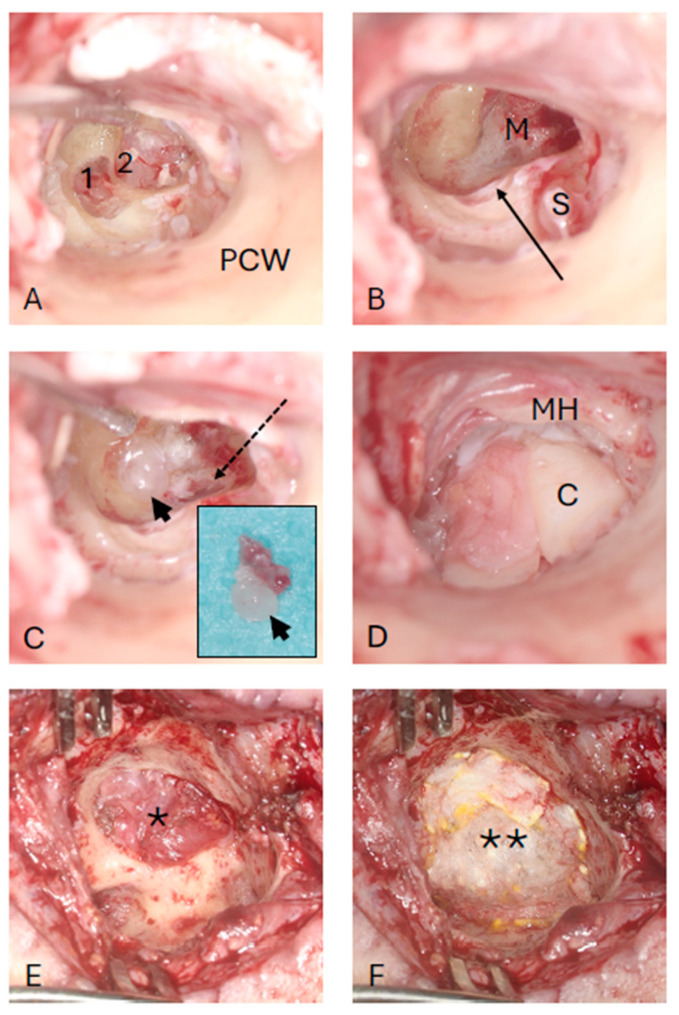
Intraoperative, transcanal view (left ear). (**A**) The cochlear capsule is partially removed. The intracochlear tumor parts are visible in the basal (1) and second (2) cochlear turn. (**B**) Removal of the intracochlear tumor parts. The vestibular entrance is blocked with soft tissue (arrow). (**C**) The tumor part from the fundus of the internal auditory canal (arrowhead) is removed with the remnants of the modiolus (insert image). The dashed arrow shows the opening of the internal auditory canal. (**D**) The defect is closed with a cartilage–perichondrium compound transplant. (**E**,**F**) Additional defect closure with a temporalis muscle patch (*) and TachoSil (**) during revision surgery with lateral petrosectomy, blind sac closure of the outer ear canal, and blockage of the tympanic opening of the Eustachian tube due to cerebrospinal fluid leak. C: cartilage M: modiolus; MH: malleus handle; PCW: posterior canal wall; S: stapes.

The possible approaches for tumor management (wait and test and scan; radiotherapy with or without CI; partial tumor removal from the cochlear scalae only and CI; or complete transcochlear tumor removal without CI) were discussed with the patient and his relatives. For tumors with extension from the cochlea through the modiolus into the IAC (transfundal IES with modiolar involvement), hearing rehabilitation with CIs is significantly more complex than for tumors confined to the inner ear if microsurgical resection is pursued [9]. Because the spiral ganglion cells in the modiolus are needed for stimulation with a CI, complete surgical tumor removal is not possible, leaving electrode insertion without tumor removal [13,16] or after partial tumor removal [14], possibly with previous radiotherapy, as options. Due to the vertigo attacks and the refusal of hearing rehabilitation with a cochlear implant, the patient underwent transcanal, transcochlear total tumor removal. The basal end of scala vestibuli, i.e., the entrance of the vestibule, was gently sealed with some soft tissue. Special care was taken through the entire procedure to not suction near or even into the vestibule. The cochlear defect was closed with a cartilage–perichondrium compound transplant as described previously [17,18] (Figure 6).

On the day after surgery, the patient reported mild subjective vertigo and therefore received physiotherapy during inpatient stay. Postoperatively, the patient reported a subjective, hardly noticeably rhinorrhea. Repeated ß_2_-Transferrin protein diagnostics, as a marker of CSF leakage, from the nose were negative (Hydrasis 2 Scan Focusing, Sebia, France). Due to a delayed mild facial nerve dysfunction (House Brackman Grade II), approximately on postoperative days 18–20, the ear canal dressing was removed. The ear canal and the tympanic membrane appeared normal for the time after surgery and ß_2_-Transferrin diagnostics from the ear canal were repeated. Due to a persisting subjective very mild clear rhinorrhea and thus clinical suspected cerebrospinal fluid (CSF) leak despite negative results for ß_2_-Transferrin protein and the delayed mild facial nerve dysfunction, revision surgery was performed with lateral petrosectomy and blind sac closure of the external auditory canal. Intraoperatively, the bony facial nerve canal looked normal but a cerebrospinal fluid leak was identified at the fundus of the IAC. The leak was sealed with temporalis muscle and a fibrinogen/thrombin sealant patch (TachoSil, Gorza Medical GmbH, Düsseldorf, Germany; Figure 6), and the cavity was obliterated with abdominal fat. The patient received a lumbar drainage and intravenous antibiotics (Ceftriaxone 2 g per day) for five days. Interestingly, the mild facial nerve dysfunction resolved already on the day of surgery. Recovery from the second surgery was uneventful and the patient reported no vertigo after this second surgery despite re-opening the site of the cochlectomy.

## 5. Follow-Up and Outcomes

**Figure 7:**
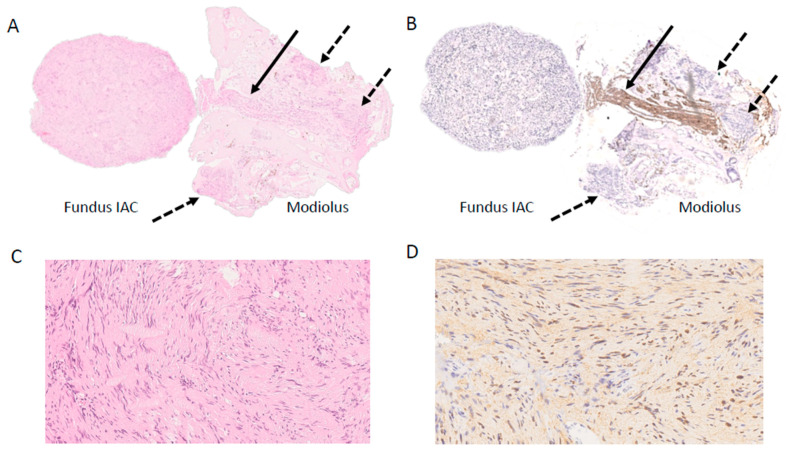
Histopathological and immunohistochemical representation. (**A**) Overview magnification of the tumor with intraosseous tumor component (dashed arrow) adjacent to a resected nerve (arrow) (H&E, 25×); (**B**) same section as A showing the resected nerve (arrow) (IHC NF200, 25×); (**C**) detailed magnification of the schwannoma (H&E, 250×); (**D**) portion of the schwannoma positive for S100 (IHC, 300×). IAC: Internal auditory canal.

Histology and immunohistochemistry showed a benign tumor (Schwannoma WHO Grade I) with a proliferative activity (Ki-67) <1% (Figure 7).

Videonystagmography, vHIT, bone-conducted oVEMPs and cVEMPs, and Romberg’s and Unterberger’s test results one week after transcochlear surgery for tumor removal and at 1-year follow-up are shown in Figure 1, Figure 3, and Figure 4. There was still some isolated spontaneous nystagmus to the right postoperatively and after 1-year follow-up (Figure 1). Postoperative caloric testing could not be performed due to blind sac closure of the ear canal. The vestibulo-ocular reflex stayed normal in all receptors (Figure 3B). The higher lateral gains had no physiological origin and were artefacts possibly due to movements of the glasses. No cervical VEMPs were present. Ocular VEMPs were measurable at 500 Hz and 1000 Hz (Figure 3), the asymmetry of 0.05 for 500 Hz and with 0.24 for 1000 Hz being within normal physiological range. Sway and rotation in Romberg’s and Unterberger’s tests, respectively, slightly deteriorated temporarily but recovered to approximately pre-surgery levels at follow-up (Figure 4).

## 6. Patient Perspective

Although the patient still noted a mild instability, he appreciated that the incapacitating vertigo attacks had subsided. The relatives noted communication problems due to the single-sided deafness. A contralateral routing of signals (CROS) hearing aid solution was declined by the patient.

## 7. Discussion

When planning the treatment of inner ear schwannomas, the expected benefits must be weighed against the risks. In cases of intact or only slightly impaired vestibular receptor function, postoperative vestibular dysfunction is one of the aspects that need to be considered. However, only few data are available on vestibular receptor function after observation, radiotherapy, or surgical treatment of intracochlear inner ear schwannomas with or without extension to the fundus of the IAC.

Due to the lack of longitudinal studies on vestibular receptor function during observation, this may indirectly be inferred from studies characterizing patient symptoms at first MRI diagnosis and various times of delay from first clinical symptoms. Several authors report vestibular receptor dysfunction also in patients with solely intracochlear IES or in IES with limited transmodiolar extension to the IAC [15,19,20].

During preoperative assessment, we observed asymmetry in the caloric excitability (with weakness at the tumor side) while vHIT for the lateral semicircular canal was normal. A dissociation of caloric and video head impulse tests with abnormal caloric response in the presence of a preserved vHIT has been suggested as an indicator of Meniere’s disease [21,22,23]. It has also been reported for delayed endolymphatic hydrops [24], vestibular schwannomas in the IAC/CPA, and for inner ear schwannomas. Blödow et al. found a higher sensitivity for caloric excitability than for vHIT (72% versus 41%) in patients with IAC/CPA tumors [25]. The dissociation between caloric response and vHIT was reported in a case of an intravestibular IES in 2007 [26]. In our own case series of intracochlear IES (without extension to the IAC), we found more patients (6 of 20, 30%) with ipsilateral weakness in the caloric responses than with reduced gain in vHIT (4 of 24, 17%) [15]. Secondary endolymphatic hydrops is present in some patients with IES [14,27,28,29]. A recent temporal bone study demonstrated that the utricular and saccular membranes often herniate into the non-ampullated limb of the horizontal canal in Menière’s disease ears on histology, which was strongly correlated with diminished caloric responses. Although in the case reported here we may hypothesize that secondary endolymphatic hydrops may have been the cause of the dissociation of the caloric response and vHIT of the lateral canal, we cannot prove this due to the lack of an MRI with adequate imaging sequence for hydrops.

Data on vestibular receptor function after radiotherapy of IES are lacking. We only found two reports on clinical symptoms, e.g., “imbalance” that had either not changed (n = 2) or improved at the one-year follow-up [30] or “vertigo that had temporarily improved but reoccurred years after radiotherapy” [31].

Very few data are available on vestibular receptor function after surgical treatment of these lesions. Plontke et al. showed in a case series of 27 consecutive patients with intracochlear IES that after partial or subtotal cochlectomy, the vestibular receptors continue to function normally in the majority of patients. This was demonstrated by specific, objective function tests for each of the peripheral vestibular receptors before and after surgery [15]. The authors stated that the mechanisms and reasons for the observed phenomenon may be explained by the anatomy of the inner ear fluid spaces (especially the ductus reuniens [32,33]), the fact that vestibular receptors are phylogenetically older than hearing receptors, and the presence of sufficient endolymph-generating cells in the vestibular labyrinth to keep the vestibular sensory organ functioning without the cochlea (for a detailed discussion, see [15]).

Laborai et al. (2022) showed that vestibular disfunction (areflexia to caloric test and reduced ipsilateral vHIT gain) of a patient with an intracochlear schwannoma treated with a “CI through tumour” strategy remained unchanged after the surgery “with just a slight increase in VOR asymmetry in the vHIT from 41% to 57% at one month and to 64% at six months” [34]. Wu et al. (2023) reported a case of endoscopic surgical removal of an intracochlear IES with “no severe episodes of vertigo at follow-up for 11 months to 2 years … but complaints of transient episodes of vertigo (1–3 s) when climbing or lifting heavy objects.” [35]. Endoscopic approaches for the surgical removal of intracochlear schwannomas with or without extension to the fundus of the IAC have also been suggested by other authors [36,37]. As no results on the postoperative functions of the vestibular receptors have been reported, the benefits in terms of functional preservation cannot yet be estimated.

Based on the single case presented here, we conclude that vestibular labyrinthine function may not only be preserved after partial or subtotal cochlectomy but also after complete cochlear removal for resection of a (limited) transmodiolar schwannoma. This further supports the observation of the robustness of the vestibular labyrinth and encourages surgical management of early-stage transmodiolar schwannomas, i.e., with limited extension to the fundus of the IAC, rather than observation.

## Data Availability

Raw data are available upon reasonable request from the corresponding author.
